# Evolution of the Seroprevalence of Pestivirus and Respiratory Viral Infections in Spanish Feedlot Lambs

**DOI:** 10.3390/ani11010160

**Published:** 2021-01-12

**Authors:** Teresa Navarro, Aurora Ortín, Oscar Cabezón, Marcelo De Las Heras, Delia Lacasta, José María González

**Affiliations:** 1 Department of Animal Pathology, University of Zaragoza & AgriFood Institute of Aragón (IA2), 50013 Zaragoza, Spain; aortin@unizar.es (A.O.); lasheras@unizar.es (M.D.L.H.); dlacasta@unizar.es (D.L.); jmgsovino@gmail.com (J.M.G.); 2 Wildlife Conservation Medicine Research Group, Department of Medicine and Animal Surgery, Autonomous University of Barcelona, 08193 Barcelona, Spain; Oscar.Cabezon@uab.cat; 3 Gabinete Técnico Veterinario S.L., 50014 Zaragoza, Spain

**Keywords:** lamb, feedlot, bovine parainfluenza virus type 3 virus, bovine respiratory syncytial virus, bovine herpesvirus type 1, pestivirus

## Abstract

**Simple Summary:**

The presence of viruses, such as bovine parainfluenza type 3, bovine herpesvirus type 1, bovine respiratory syncytial virus and pestiviruses, has been widely demonstrated in sheep in the past, but it is unknown how these infections may affect the lambs during their fattening period under commercial conditions. In the present study, the exposure to the aforementioned viruses was studied in 120 feedlot lambs throughout the whole fattening period. Seroprevalences were measured by enzyme-linked immunosorbent assays (ELISA), relating their presence to health and production parameters in the studied lambs. During the studied period, the lambs seroconverted against 3 out of the 4 viruses analysed and only 10.8% of the lambs were seronegative for all the tested viruses throughout the entire study. Finally, no associations were found between the seroconversion to one or more viruses and the presence of respiratory clinical signs or lung lesions at the slaughterhouse. In addition, no disturbances were observed in the performance of the lambs, except in the case of pestivirus infections, for which the presence of antibodies in the animals was associated with reduced final weight at the end of the fattening period.

**Abstract:**

The presence of respiratory viruses and pestiviruses in sheep has been widely demonstrated, and their ability to cause injury and predispose to respiratory processes have been proven experimentally. A longitudinal observational study was performed to determine the seroprevalence of bovine parainfluenza virus type 3 (BPIV-3), bovine respiratory syncytial virus (BRSV), bovine herpesvirus type 1 (BHV-1) and pestiviruses in 120 lambs at the beginning and the end of the fattening period. During this time, the animals were clinically monitored, their growth was recorded, and post-mortem examinations were performed in order to identify the presence of pneumonic lesions in the animals. Seroconversion to all viruses tested except BHV-1 was detected at the end of the period. Initially, BPIV-3 antibodies were the most frequently found, while the most common seroconversion through the analysed period occurred to BRSV. Only 10.8% of the lambs showed no detectable levels of antibodies against any of the tested viruses at the end of the survey. In addition, no statistical differences were found in the presentation of respiratory clinical signs, pneumonic lesions nor in the production performance between lambs that seroconverted and those which did not, except in the case of pestiviruses. The seroconversion to pestiviruses was associated with a reduction in the final weight of the lambs.

## 1. Introduction

Ovine respiratory complex (ORC) is the second leading cause of disease in Spanish lamb feedlots, after coccidiosis, and is the primary cause of death in all types of farms, seasons, regions, breeds and weights in the country [1]. ORC is caused by several agents, but the five microorganisms present in more than two-thirds of the isolates are *Mannheimia haemolytica*, *Pasteurella multocida*, *Bibersteinia trehalosi*, *Mycoplasma* spp. and *Escherichia coli* [1]. However, the involvement of viral agents in this disease has not been clarified in lambs.

It is well known the role of respiratory viruses such as bovine parainfluenza virus type 3 (BPIV-3), bovine respiratory syncytial virus (BRSV), bovine herpesvirus type 1 (BHV-1) and pestiviruses in the development of bovine respiratory disease (BRD). In sheep and lambs, evidence of natural infection by these viruses has been reported by several authors in different geographic locations [2,3,4,5]. In addition, experimental infections performed in different lamb models have proven the ability of respiratory viruses to cause disease in ovines [6,7,8,9,10,11]. Similarly, despite respiratory lesions not being the main feature of pestivirus infections, lung damage has been noted under certain conditions in cattle [12,13,14] but also in sheep in the case of experimental infections with border disease virus (BDV) [15]. Likewise, other pestiviruses different than BDV, such as bovine viral diarrhoea virus (BVDV) have also demonstrated being able to infect lambs [14]. The susceptibility to some of these viruses seems to be age-dependent, decreasing progressively as the age of the lamb increases [15,16]. Furthermore, under experimental conditions, the consequences derived from these infections in lambs tend to be mild unless they are combined with a bacterial infection, especially by Pasteurellaceae [11,15,16,17]. The most common clinical signs associated with respiratory viruses are nasal discharge, tachypnoea and fever [16]. However, transient immune suppression has been described as one of the main pathogenic effects associated with these viral infections in cattle [18,19,20], also reported in ovines [5,21].

The aim of this study was to elucidate the importance of these viruses in feedlot lambs under natural conditions through the observation of the evolution of seroconversions during the fattening period. Health and production data from this period were also analysed, evaluating the influence of the viral infections on these parameters that have a relevant economic impact.

## 2. Materials and Methods 

A longitudinal observational study was conducted in a lamb feedlot located in Aragón (North-Eastern Spain). One hundred and twenty Rasa Aragonesa clinically healthy fattening male lambs without clinical signs of disease were randomly selected throughout all seasons in two years, distributed in 8 groups of 15 animals each. Lambs were recruited at weaning age (40–50 days old) with 12–15 kg of live weight. All the studied animals were individually identified and tagged at the farm of origin. On arrival at the feedlot, the lambs were classified and stocked in groups mixed with other animals at a maximum density of 1.67 lambs/m^2^, and after six weeks of fattening (82–92 days old), they were sent to the slaughterhouse. Blood samples without anticoagulant were taken by jugular venipuncture from all the lambs at two different moments: on the farm of birth, just before the transport to the feedlot, and at the end of the fattening period, prior to being transported to the slaughterhouse. Immediately after collection, the samples were refrigerated and sent to the laboratory, processed to obtain sera, aliquoted and stored at −20 °C until analysis. The animals were monitored daily for clinical signs of disease throughout the study period by specialised personnel, the same clinician throughout the study, and their weights were registered weekly. Once in the slaughterhouse, the carcasses’ weights were recorded after evisceration. Finally, the viscera were submitted to the pathology department of the Veterinary Faculty of Zaragoza for post-mortem examination and identification of gross pneumonic lesions. 

Detection of antibodies against the different viruses was performed by means of commercially available enzyme-linked immunosorbent assay (ELISA) kits: IDEXX PI-3 Ab Test for BPIV-3 antibody detection (sensitivity and specificity of 98.9% and 75.0%, respectively, according to the information of the manufacturer’s validation data report); IDEXX IBR gB X3 Ab for BHV-1 antibodies (reported sensitivity and specificity of 100% and 99.8%, respectively); IDEXX BVDV p80 Ab in the case of pestiviruses (97.6% sensitivity and 97.3% specificity), which detects p80 polypeptide, common to most pestiviruses, including BDV, BVDV and classical swine fever virus (CSFV) (IDEXX Laboratories, Montpellier, France); and CIVTEST Bovis BRSV/VRS for BRSV (75.7% sensitivity and 93.9% specificity) (Laboratorios Hipra S.A., Amer, Spain), according to the manufacturer’s instructions. The serological results were categorised as positive or negative, and seroconversion was defined as a change in the serological status (final vs. initial), except in the case of BPIV-3 determinations, that allowed allocation of each animal into four different categories according to their optical densities and based on cut-points provided by the manufacturer. The categories provided were: (1) 20–40%, (2) 40–60%, (3) 60–80% and (4) 80–100% of seropositivity. According to this, samples beyond 20% of seropositivity were considered positive, but only differences of at least two increasing categories between final and initial status were indicative of seroconversion.

Statistical analysis was conducted through the statistical package IBM SPSS Statistics v.22.0 (SPSS Inc., Chicago, IL, USA). The association between seroconversion to the different viruses, clinical signs of respiratory disease and pneumonic lesions identified at post-mortem study according to the final serological status was evaluated by Chi-square test, while Fisher’s exact test was used when the sample size for a variable was below 5. The differences in the average daily gain were analysed by means of non-parametric tests for two independent samples with Mann–Whitney U test. The final weights were analysed by means of the univariated general linear model procedure according to the final serological status and season as fixed factors and covariated by initial weight of the lambs. These procedures were performed for each virus individually and also for all of them collectively.

## 3. Results

Seropositivity was evidenced for all tested viruses, except for BHV-1, for which all the animals were seronegative in the two samplings, either in the feedlot or on the farm of origin (Table 1). At weaning, BPIV-3 antibodies were the most frequently detected, followed by pestivirus and BRSV (Table 1). Moreover, multiple infections were found in 19 of the 120 analysed animals, with 14 lambs positive for BPIV-3 and BRSV, 1 for BPIV-3 and pestivirus, 1 for BRSV and pestivirus, and 3 for all of them.

At the end of the fattening period, only 13 out of the 120 studied lambs (10.8%) remained seronegative to all the viruses tested. During this period, the highest level of seroconversion occurred to BRSV. Thus, 43 lambs increased their antibody levels reaching a positive status by the end of the fattening period, while 15 positive lambs at weaning became seronegative at the at the end of the same period. Similar results were observed with BPIV-3, with 30 showing seroconversion at the end of the fattening period, although of these animals, 8 were already positive for this virus at the initial sample and increased their antibody titre by the end of the study and additionally, 11 lambs became seronegative at the final sample. Regarding pestivirus, 13 and 6 lambs changed their status to negative or positive at the end of the study, respectively.

Finally, an association was observed between BRSV and BPIV-3 exposure (*p* = 0.006) and the lambs that seroconverted to BRSV presented higher risk to seroconvert to BPIV-3 (RR = 2.342).

The most frequently reported clinical signs observed in the lambs throughout the studied period were: dyspnoea, conjunctivitis, nasal discharge and coughing. These symptoms were present alone or in combination in 9 of the lambs that seroconverted to BRSV, in 8 of those seroconverting to BPIV-3 and 1 out of 6 lambs that seroconverted to pestiviruses. Nevertheless, statistically significant associations between seroconversion to any of the viruses studied and the detection of respiratory clinical signs in the animals were not observed in this investigation (Table 2). Similarly, the identification of gross pneumonic lesions at post-mortem examination never revealed any statistical relationship with seroconversion to any of the viruses.

Regarding the repercussions of these viral infections on performance, the average daily gain (ADG), the final weight and the carcass yield were similar for all groups of animals, and no statistically significant associations were found between them except for lambs that seroconverted to pestiviruses (*p* < 0.05). These animals showed a mean final live weight 2.730 kg lower (23.393 vs. 26.123) compared with those that did not evidence exposure (*p* < 0.05).

## 4. Discussion

All the viruses studied in the present study are associated with pathological effects in cattle while there is also evidence of their infection in sheep. Among the studied viruses, BHV-1 seems to be the least common in ovines [2,4], although sheep exposed to BHV-1 have demonstrated not always exhibiting a detectable humoral immune response or virus excretion [8]. For these reasons, the transmission of this virus in small ruminants seems to be infrequent [8].

Seroprevalences for BPIV-3, BRSV and pestiviruses found in the present study are similar to those reported in other works conducted in sheep, even though most of them were performed at flock level and under different production systems. In general, the literature indicates widespread BPIV-3, with seroprevalences higher than 70% in various reports [2,22,23], although low rates have been also reported [4,24]. Values given by other authors are in accordance with those obtained in this work (38.3%) regarding BRSV and tend to be slightly lower to those recorded for BPIV-3 (87.5%), which can be found in a wide range (24%–71.5%) [2,22,24,25]. By contrast, pestivirus antibodies seem to be at lower values in most of the reports from different countries, even below 1% [2,26]. However, in the case of Spanish fattening lambs, the reported seroprevalences range from 13.8% to 28.6% [5,27], as the infection is considered endemic in the country and, consequently, higher seroprevalences can be found [28].

For all these viruses, there were animals with detectable levels of antibodies at weaning time that became seronegative by the end of the studied period. Maternally derived antibodies have shown to remain detectable in lambs for 2–5 months, in the case of BPIV-3 [29], and according to the observations performed in cattle, for 3–4 months and 1–4 months in the case of BRSV [30] and pestivirus, respectively [31,32]. However, the dynamics of the infection throughout the study have differed between the viruses. Whereas increasing levels of antibodies to BPIV-3 and BRSV were detected in the lambs between initial and final samplings, seroconversion to pestiviruses was not frequent in the feedlot, despite the initial seroprevalence revealing a significant presence of the virus on the farms of origin. In this sense, in a survey performed in feedlot lambs in conditions very similar to the present work, the authors observed that the majority of pestivirus infections occurred 27 days after the arrival of the lambs at the feedlot. In addition, only 2 of the 17 animals in which the virus was previously detected had seroconverted at the end of the fattening period, 42 days later [5]. As a consequence of the long time span for detectable seroconversion in response to pestivirus infection [33], the viability of antibody detection as a technique for the investigation of the dynamics of this particular infection is reduced in Spanish fattening lambs due to their short feedlot stay.

Despite the fact that the pathogenicity of all four studied viruses has been demonstrated through experimental infections in several lamb models [9,10,15,16], clear pathologic consequences of the infection were not observed in the present survey. In cattle, a modest increase in the risk of suffering from BRD after seroconversion to each virus separately has been observed, which progressively increases with multiple viral exposures, underlining the importance of concurrent infections [34]. In the present study, concurrent infections have been observed; however, no apparent pathological consequences were detected. Finally, lambs which had seroconverted to pestiviruses during the fattening period showed a statistically-significant lower mean final live weight compared to those that did not, although these results should be interpreted with caution due to the reduced sample size. However, similar observations were made by González et al. in feedlot lambs [4]. Likewise, in cattle, the milder effect of BPIV-3 and BRSV compared to pestiviruses infections was observed through the reduced weight of the affected animals at the end of the fattening period [35].

## 5. Conclusions

The exposure of lambs to BRSV, BPIV-3 and pestiviruses on the farms, but also in the feedlot, has been demonstrated in this study, while no proof of BHV-1 infection was determined. However, despite the presence of these viruses and their ability to cause disease under experimental conditions, the detrimental effects derived from their exposure in natural conditions were not observed, with the exception of pestivirus infection, which revealed a significant adverse effect on the final weight of the studied lambs.

## Figures and Tables

**Table 1:** Apparent seroprevalence to the bovine respiratory syncytial virus (BRSV), bovine parainfluenza virus type 3 (BPIV-3) and pestivirus in 120 lambs at weaning (40–50 days old), at the end of the fattening period (42 days later) and the total seroconversion that occurred during this period.

	BRSV		BPIV-3		Pestivirus		Total	
	No. Lambs	%	No. Lambs	%	No. Lambs	%	No. Lambs	%
Seroprevalence at weaning	18	15.0 (8.6%, 21.4%) ^a^	70	58.3 (49.5%, 67.2%) ^a^	29	24.2 (16.5%, 31.8%) ^a^	85	70.8
Seroprevalence at the end of fattening period	46	38.3 (29.6%, 47.0%) ^a^	105	87.5 (81.6%, 93.4%) ^a^	22	18.3 (11.4%, 25.3%) ^a^	107	89.1
Seroconversion during fattening period	43	35.8	30	25.0	6	5.0	58	48.3

^a^ 95% Confidence Interval.

**Table 2:** Differences in the presentation of clinical signs, pneumonic lesions, average daily gain (ADG) and carcass yield in 120 lambs during the fattening period, according to seroconversion to bovine parainfluenza virus type 3 (BPIV-3), bovine respiratory syncytial virus (BRSV) or pestiviruses.

Seroconversion to Any Virus (BPIV-3, BRSV, Pestiviruses)		No Seroconversion *n* = 62	Seroconversion *n* = 58	*p*
Respiratory clinical signs	No. Lambs	8	14	0.112
	%	12.9	24.1	
Lung consolidated lesions	No. Lambs	19	19	0.807
	%	30.6	32.8	
Average daily gain (kg/d)		0.305 ± 0.0562	0.306 ± 0.0685	0.646
Carcass yield (%)		45.5 ± 2.14	45.0 ± 2.26	0.166
Final weight (kg)		26.129 ± 3.7413	26.245 ± 3.5701	0.799

## Data Availability

The data presented in this study are available on request from the corresponding author. The data are not publicly available due to privacy reasons.
